# Multiple factors interact to produce responses resembling spectrum of human disease in *Campylobacter jejuni *infected C57BL/6 IL-10^-/- ^mice

**DOI:** 10.1186/1471-2180-9-57

**Published:** 2009-03-18

**Authors:** Julia A Bell, Jessica L St Charles, Alice J Murphy, Vijay AK Rathinam, Anne E Plovanich-Jones, Erin L Stanley, John E Wolf, Jenna R Gettings, Thomas S Whittam, Linda S Mansfield

**Affiliations:** 1 Comparative Enteric Diseases Laboratory, National Food Safety and Toxicology Center, Michigan State University, East Lansing, Michigan, 48824, USA; 2 Department of Food Science and Human Nutrition, National Food Safety and Toxicology Center, Michigan State University, East Lansing, Michigan, 48824, USA; 3 Department of Microbiology and Molecular Genetics, National Food Safety and Toxicology Center, Michigan State University, East Lansing, Michigan, 48824, USA

## Abstract

**Background:**

*Campylobacter jejuni *infection produces a spectrum of clinical presentations in humans – including asymptomatic carriage, watery diarrhea, and bloody diarrhea – and has been epidemiologically associated with subsequent autoimmune neuropathies. This microorganism is genetically variable and possesses genetic mechanisms that may contribute to variability in nature. However, relationships between genetic variation in the pathogen and variation in disease manifestation in the host are not understood. We took a comparative experimental approach to explore differences among different *C. jejuni *strains and studied the effect of diet on disease manifestation in an interleukin-10 deficient mouse model.

**Results:**

In the comparative study, C57BL/6 interleukin-10^-/- ^mice were infected with seven genetically distinct *C. jejuni *strains. Four strains colonized the mice and caused disease; one colonized with no disease; two did not colonize. A DNA:DNA microarray comparison of the strain that colonized mice without disease to *C. jejuni *11168 that caused disease revealed that putative virulence determinants, including loci encoding surface structures known to be involved in *C. jejuni *pathogenesis, differed from or were absent in the strain that did not cause disease. In the experimental study, the five colonizing strains were passaged four times in mice. For three strains, serial passage produced increased incidence and degree of pathology and decreased time to develop pathology; disease shifted from watery to bloody diarrhea. Mice kept on an ~6% fat diet or switched from an ~12% fat diet to an ~6% fat diet just before infection with a non-adapted strain also exhibited increased incidence and severity of disease and decreased time to develop disease, although the effects of diet were only statistically significant in one experiment.

**Conclusion:**

*C. jejuni *strain genetic background and adaptation of the strain to the host by serial passage contribute to differences in disease manifestations of *C. jejuni *infection in C57BL/6 IL-10^-/- ^mice; differences in environmental factors such as diet may also affect disease manifestation. These results in mice reflect the spectrum of clinical presentations of *C. jejuni *gastroenteritis in humans and contribute to usefulness of the model in studying human disease.

## Background

*Campylobacter jejuni *is a major cause of food-borne gastroenteritis worldwide. In addition to causing disease in humans, this microorganism can colonize a variety of domestic animals, common and exotic pets, and domestic and wild birds; some of these alternate hosts experience disease [1,2]. Successful experimental colonization of several mouse strains with *C. jejuni *has been reported, but disease does not occur unless mice are immunodeficient or wild type mice are experimentally manipulated [3].

Clinical presentation of campylobacteriosis in human patients in industrialized countries usually varies from mild watery diarrhea to severe bloody diarrhea; in developing countries, milder diarrhea and asymptomatic infections are also seen [1,2]. Bacteremia can occur. Antecedent *C. jejuni *infection has been associated with the development of reactive arthritis and the autoimmune neuropathies Guillain Barré and Miller Fisher Syndromes. Disease expression in humans is likely the result of complex interactions between pathogen genetic properties, host genetic properties, host physiological state and immune response, and the host intestinal microbiota [2,4]. Environmental factors such as host diet may affect one or more of these factors; diet variables may act through mechanisms such as modulation of the host immune system by fatty acids or alteration of the composition of the complex microbial populations of the lower GI tract [5].

*C. jejuni *is a genetically variable organism [6]. Over 3000 sequence types are cataloged in the *Campylobacter jejuni *Multi Locus Sequence Typing (MLST) database [7], and numerous studies employing other typing methods such as restriction fragment length polymorphisms (RFLP) in an array of genes, amplified fragment length polymorphisms, and microarray-based comparisons of entire genomes have consistently revealed substantial genetic variation [8-13]. Furthermore, genetic variation has been documented in a number of virulence determinants, including genes involved in motility, iron metabolism, toxin synthesis and secretion, adherence to and invasion of eukaryotic cells, and capsule and lipo-oligosaccharide (LOS) synthesis [14-23]. Genetic variation affecting gene expression has been directly linked to *in vivo *variation in pathogenicity of two otherwise very similar strains from poultry [24].

*C. jejuni *also possesses mechanisms that could be expected to generate genetic diversity *in vivo*. MLST data, based on analysis of DNA sequences of genes for proteins of central metabolic pathways, have been used to deduce that recombination occurs in natural *C. jejuni *populations, both within *C. jejuni *and between *C. jejuni *and the closely related *C. coli *[25-27], although estimates of the frequency of recombination vary with the method of analysis used and/or with the type of population studied. De Boer *et al*. [28] demonstrated *in vivo *genetic exchange between *C. jejuni *strains coinfecting chickens. Phase variation via slip-strand mutagenesis in homopolymeric tracts has been demonstrated in a motility-related gene [29], a capsular synthesis gene [18], and a lipo-oligosaccharide (LOS) synthesis gene [17]. In the latter case, phase variation results in switching the genes encoding the LOS structure between forms mimicking GM1 or GM2 gangliosides found in neural tissue; it is thought that the reaction with neural tissue of autoimmune antibodies directed against LOS molecules that mimic neural gangliosides underlies the development of Guillain-Barré and Miller Fisher syndromes. Prendergast *et al*. demonstrated *in vivo *phase variation in the LOS genes in experimentally infected human volunteers [30].

Evolutionary changes in pathogenicity of pathogens (*i. e*., increase or decrease in virulence) are thought to be the result of trade-offs between host mortality and probability of transmission to a new host, although immunopathology resulting from damage caused by the immune response may modulate the selective process [31-33]. Both host and pathogen genetic factors may be important in the evolutionary process [34]. Serial passage experiments that explore virulence evolution have usually resulted in increased pathogen-induced damage to the host [35,36]. A few serial passage experiments have been conducted with *C. jejuni*. Fernández *et al*. [37] showed that serial intraperitoneal passage in mice of ten *C. jejuni *strains that could not invade HEp2 cells in culture restored and then enhanced this ability, but pathogenicity of the passaged strains in intestinal infections of mice was not determined. Chickens are commensally colonized by *C. jejuni *and are an important reservoir for human infection. Ringoir and Korolik [38] showed that serial passage of four *C. jejuni *strains in chickens reduced the minimum infectious dose required for colonization. Jones *et al*. [39] showed that passage of a poorly motile variant of *C. jejuni *11168 in chickens increased the ability of this strain to colonize and persist in chickens; this change was accompanied by an increase in motility.

Development of a murine model of *C. jejuni *infection in which C57BL/6 IL-10^+/+ ^mice are colonized by *C. jejuni *11168 while C57BL/6 IL-10^-/- ^mice are both colonized and experience enteritis allowed us to explore the relationship between genetic variation in *C. jejuni *and disease expression in a model in which host genetic factors are close to identical and host environmental factors can be either standardized or varied in a controlled way [40]. Our first hypothesis was that *C. jejuni *strains from humans, chickens, and cattle vary in their ability to colonize and cause enteritis in C57BL/6 IL-10^-/- ^mice. Our second hypothesis was that serial passage of *C. jejuni *strains in C57BL/6 IL-10^-/- ^mice would increase the ability of the passaged strains to cause enteritis in mice. The data obtained supported both of these hypotheses. Furthermore, during the course of these experiments, it became apparent that dietary factors can also influence disease expression in this mouse model.

## Results

Five experiments are reported here. Experiment 1 comprised genetic comparisons of seven *C. jejuni *strains by multilocus sequence typing and restriction fragment polymorphism analysis of known and putative virulence loci. Experiment 2 comprised four serial passages of each of five *C. jejuni *strains in C57BL/6 IL-10^-/- ^mice. The final passage in experiment 2 also included (1) a comparison of passaged strains with unpassaged *C. jejuni *11168 and (2) a comparison of mice infected with unpassaged *C. jejuni *11168 kept on an ~12% fat breeder diet and mice infected with unpassaged *C. jejuni *11168 experiencing a transition from the ~12% fat breeder diet to an ~6% fat maintenance diet just prior to inoculation. Experiment 3 was suggested by the results of experiment 2 and comprised a whole ORF microarray comparison of the gene content of *C. jejuni *strains 11168 and NW. Experiment 4 was also suggested by the results of experiment 2 and comprised a short term (48 hour) infection study of passaged and unpassaged *C. jejuni *11168 strains to determine whether there were any differences in ability of the strains to cause enteritis immediately after infection. Experiment 5 was suggested by the results of the dietary comparison included in the final passage of experiment 2 and comprised a comparison of mice infected with unpassaged *C. jejuni *11168; mice were kept on the ~12% fat diet throughout the experiment, were kept on the ~6% fat diet throughout the experiment, or were subjected to a transition from the ~12% fat diet to the ~6% fat diet just prior to inoculation.

### *C. jejuni *strains used in this study were genetically variable in both housekeeping genes and virulence determinants (experiment 1)

**Figure 1:**
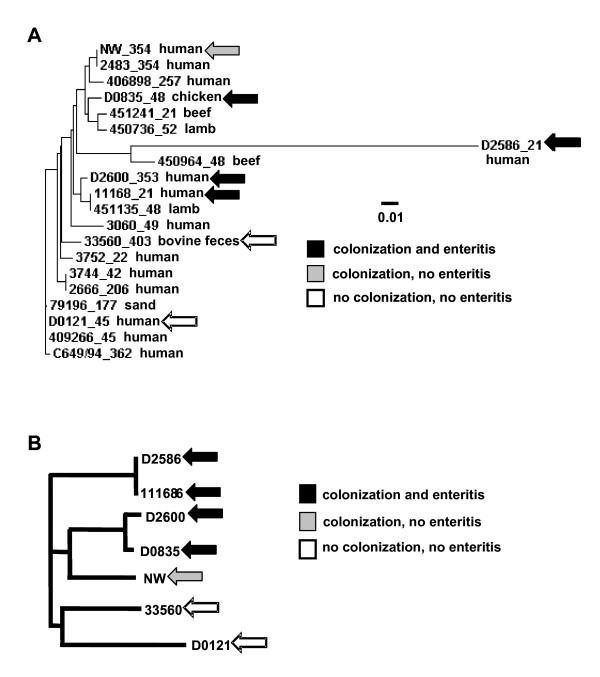
**Neighbor-joining trees based on MLST data and RFLP data for putative virulence determinants (experiment 1)**. Panel A, MLST; panel B, virulence gene RFLP. The MLST tree also includes MLST sequences for reference strains of major clonal complexes established by Wareing et al. [42] obtained from the *Campylobacter jejuni *MLST database [7]. Each strain name is followed by the number of the clonal complex to which that strain belongs and the source from which it was isolated. The two most distantly related strains in the virulence gene RFLP analysis, D0121 and D2600, had a Jaccard similarity coefficient of 0.45.

The seven *C. jejuni *strains used in this study are listed in Table 1. They represent six MLST sequence types in six clonal complexes and were chosen in part so as to span the genetic diversity of the strains characterized by MLST by Sails *et al*. [41]. An MLST-based neighbor-joining tree displaying the genetic relationships of these strains to each other and to reference strains for the major *C. jejuni *clonal complexes is shown in Figure 1A; the tree includes MLST sequences for reference strains of major clonal complexes established by Wareing et al. [42]. Sequences for the reference strains and all strains used in this study except strain NW were obtained from the *Campylobacter jejuni *MLST database [7]. MLST typing of strain NW was carried out in our laboratory (GenBank accession numbers FJ361183 through FJ361189) and the clonal complex determined using the *Campylobacter jejuni *MLST database. The two strains that were unable to colonize the mice at levels detectable by culture (see below) both clustered at a distance from each other and from the colonizing strains; the colonizing strains clustered together with the strain that colonized but did not produce disease.

**Table 1:** Characteristics of *Campylobacter jejuni *strains used in this study.

*C. jejuni *strain	Species of origin, disease status, location	Source	MLST sequence type (clonal complex)
11168	Human disease UK	American Type Culture Collection	21 (ST 43)
D2586	Human disease UK	Centers for Disease Control	21 (ST 43)
D2600	Human disease USA	Centers for Disease Control	353 (ST 452)
D0835	Chicken carrier USA	Centers for Disease Control	48 (ST 429)
NW	Human disease Africa	Sparrow Hospital, Lansing, MI	354 (ST 354)
33560^T^	Bovine carrier USA	American Type Culture Collection	403 (ST 403)
D0121	Human unknown Canada	Centers for Disease Control	45 (ST 45)

The seven strains were assayed by polymerase chain reaction (PCR) with gene-specific primers for the presence of a number of known or putative virulence determinants for which presence/absence variation had previously been documented in epidemiological studies (Table 2; [21,22]). None of the strains were PCR-positive for the plasmid-borne *vir*B11 gene; as a control for the PCR assay, we verified the presence of the *vir*B11 gene in strain 81–176, which carries the pVir plasmid [43]. Strains D2600, D0835, and NW were PCR-negative for the *iam *marker; strain D2600 was also PCR-negative for the *wla*N gene. Restriction fragment polymorphism (RFLP) analysis was performed on PCR products of the *fla*A, LOS, *cdt*ABC, *ceu*E, *pld*A, *cia*B, *dna*J, and *cgt*B genes of these strains. The resulting banding patterns were used to generate the neighbor-joining tree shown in Figure 1B. The two strains that were unable to colonize the mice at levels detectable by culture again clustered at a distance from each other and from the colonizing strains. Strains 11168 and D2586 were identical in the RFLP analysis of virulence-associated loci but rather different in MLST. Similarly, strains D2600 and D0835 had very similar RFLP patterns but appeared in different MLST clusters.

**Table 2:** Virulence determinants detected by gene-specific PCR assay.

		Strain							
Gene	Function	11168	D2586	D2600	D0835	NW	33560	D0121	Citation
***cad*F**	adherence	**+**	**+**	**+**	**+**	**+**	**+**	**+**	[16]
***cdt*ABC**	cytolethal distending toxin	**+**	**+**	**+**	**+**	**+**	**+**	**+**	[21]
***ceuE ***	iron metabolism/ siderophore	**+**	**+**	**+**	**+**	**+**	**+**	**+**	[15]
***cgt*B**	β-1,3-galactosyl-transferase	**+**	**+**	**+**	**+**	**+**	**+**	**+**	[15]
***cia*B**	internalization	**+**	**+**	**+**	**+**	**+**	**+**	**+**	[22]
***dna*J**	heat stress	**+**	**+**	**+**	**+**	**+**	**+**	**+**	[22]
***fla*A**	motility	**+**	**+**	**+**	**+**	**+**	**+**	**+**	[14]
***iam*B**	adhesion/invasion	**+**	**+**	**-**	**-**	**-**	**+**	**+**	[19]
**LPS operon**	lipopolysaccharide	**+**	**+**	**+**	**+**	**+**	**+**	**+**	[20]
***pld*A**	phopholipase	**+**	**+**	**+**	**+**	**+**	**+**	**+**	[22]
***rac*R**	colonization	**+**	**+**	**+**	**+**	**+**	**+**	**+**	[22]
***wla*N**	β-1,3-galactosyl-transferase	**+**	**+**	**-**	**+**	**+**	**+**	**+**	[17]
									
**Other loci thought to be involved in virulence**									
									
***lux*S**	global regulator	**+**	**+**	**+**	**+**	**+**	**+**	**+**	This study
***vir*B11 (plasmid)**	type IV secretion	**-**	**-**	**-**	**-**	**-**	**-**	**-**	[43]

### *C. jejuni *strains differed in their ability to colonize and cause enteritis in C57BL/6 IL-10^-/- ^mice in the initial passage of experiment 2 (serial passage experiment)

**Figure 2:**
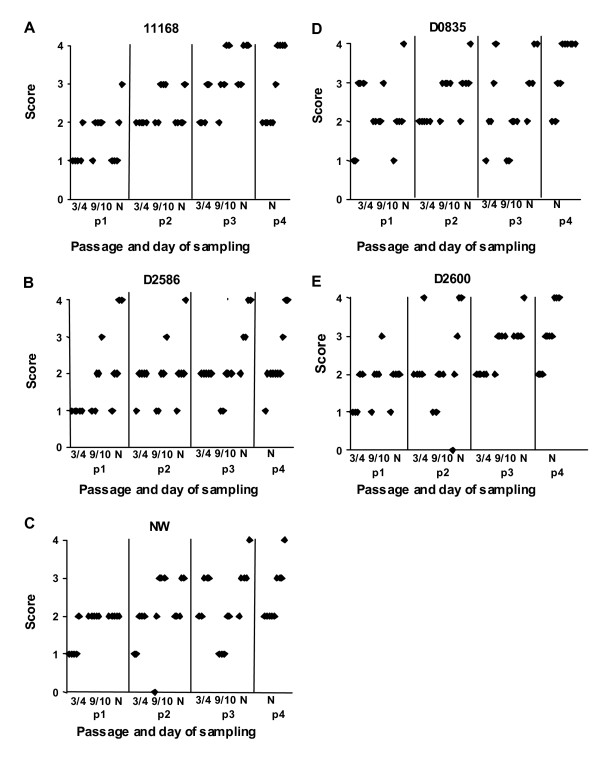
**Culturable fecal populations of colonizing *C. jejuni *strains in C57BL/6 IL-10^-/- ^mice (experiment 2)**. Levels of growth on TSA-CVA agar medium were scored on a scale of 0 to 4 (0, no colonies; 1, ≤ ~20 colonies; 2, ~20–200 colonies; 3, ≥ ~200 colonies; 4, confluent growth). *C. jejuni *was not recovered by culture from mice inoculated with tryptose soya broth or with non-colonizing strains 33560 and D0121 at any time. Each point represents an individual mouse.

Mice were infected with total doses of ~1 × 10^10 ^cfu *C. jejun*i, housed individually for 30–35 days, and then euthanized and necropsied as previously described [40]. *C. jejuni *cells in wet mounts of all suspensions used to inoculate mice were highly motile. Mice were evaluated twice daily for clinical signs of disease and euthanized promptly if severe clinical signs were observed. Fecal samples were taken on days 3 or 4, 9 or 10, and at necropsy and spread on medium selective for *C. jejuni *(Figure 2). Additional detailed colonization data are presented in Additional file 1 (Additional file 1, Table S1). As shown in the summary in Table 3, five of the seven strains were able to colonize the mice;*C. jejuni *could be cultured from the feces of 5/5 mice inoculated with strains 11168, D0835, D2586, D2600, and NW on all days of sampling and from tissue and fecal samples obtained at necropsy (Figure 2; Additional file 1, Table S1). Strains 33560 and D0121 were never recovered by culture from fecal samples taken during the course of infection (data not shown) or from tissues or feces collected at necropsy (Additional file 1, Table S1). Strain 33560 DNA was present at low levels in multiple tissues collected at necropsy as shown by PCR assay for the *C. jejuni gyr*A gene [44] performed on DNA extracted from tissues, but strain D0121 was only weakly detected in two tissue samples by PCR assay (Additional file 1, Table S1). Cultures were verified using the same PCR assay.

**Table 3:** Initial ability of *C. jejuni *strains to colonize and cause enteritis in C57BL/6 IL-10^-/- ^mice.

*C. jejuni *strain	*C. jejuni *detectable by culture; culture verified by PCR	*C. jejuni *detectable by PCR; not detectable by culture	Number of mice having gross pathology*	Number of mice having histopathology (score ≥ 10)**
**11168**	5/5 mice multiple tissues	NA***	1/5	1/5
**D2586**	5/5 mice multiple tissues	NA	2/5	1/5
**D2600**	5/5 mice multiple tissues	NA	2/5	1/5
**D0835**	5/5 mice multiple tissues	NA	2/5	1/5
**NW**	5/5 mice multiple tissues	NA	2/5	0/5
**33560 (D0133)**	0/5 mice multiple tissues	5/5 mice multiple tissues	0/5	0/5
**D0121**	0/5 mice multiple tissues	2/5 mice weak band single tissue	0/5	0/5

* The following gross pathological changes were recorded: enlarged ileocecocolic lymph nodes, thickened colon wall, and presence of blood in the intestinal lumen.

** See Mansfield et al. [40] for details of the scoring system used.

*** NA, not applicable.

**Figure 3:**
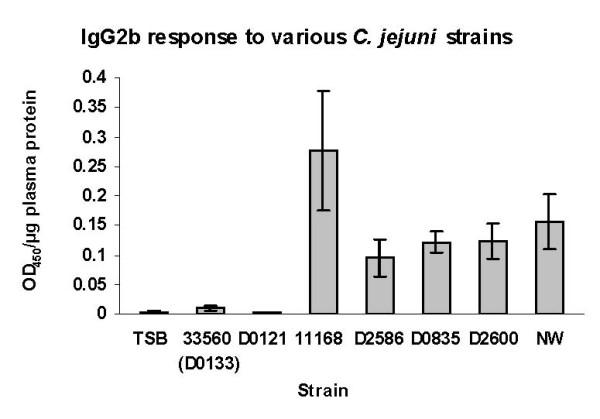
**Plasma levels of anti-*C. jejuni *IgG2b produced by C57BL/6 IL-10^-/- ^mice (first passage, experiment 2)**. Strains were non-adapted; each bar represents the average of five mice; whiskers indicate standard error. TSB, sham inoculated control mice.

Further evidence that strains 33560 and D0121 were unable to persistently colonize the mice is provided by the fact that while all five of the colonizing strains evoked circulating IgG2b antibody responses, the two non-colonizing strains evoked little or no antibody as shown in Figure 3. IgG2b accounts for the bulk of the antibody response of C57BL/6 IL-10^-/- ^mice to *C. jejuni *[40].

All five colonizing strains were able to cause some gross pathological changes observed at necropsy, including enlarged ileocecocolic lymph nodes, thickened colon wall, and bloody contents in the intestinal lumen (Table 3). The most common gross pathological change was the occurrence of enlarged ileocecocolic lymph nodes. In previous experiments, in about one-third of C57BL/6 IL-10^-/- ^mice infected with non-adapted *C. jejuni *11168, the only gross pathological change observed was an enlarged ileocecocolic lymph node and the histopathology score at the ileocecocolic junction was ≤ 10 (Grade 0). Four of the five colonizing strains were able to produce histopathological changes at the ileocecocolic junction that resulted in a histopathology score ≥ 10 in at least one mouse in the initial passage (Table 3). (See [40] for details of the scoring system used. Briefly, the intestinal lumen and three layers of the intestinal wall (mucosa, lamina propria, and submucosa) were evaluated separately for indicators of inflammation such as excess mucus, tissue hyperplasia, tissue architecture and integrity, infiltration of monocytes and neutrophils, edema, fibrosis, and vasculitis. Characters contributed to a score that ranged from 0 to 44; scores less than 10 were considered normal.)

### Three *C. jejuni *strains caused more severe enteritis following serial passage (experiment 2, serial passage experiment)

For colonizing *C. jejuni *strains, the initial results described above were obtained in the first of four serial passages. For subsequent passages, *C. jejuni *growth from cecal tissue of each individual mouse was harvested and used as the inoculum for the next serial passage. All of the *C. jejuni *growth from the ceca of all mice inoculated with a single strain was pooled to produce the inoculum for the next passage. In passages 1 through 3, five mice were inoculated with each *C. jejuni *strain; ten mice were inoculated with each strain in passage 4.

As noted below (Materials and Methods), in this series of experiments, mice in the first passage were inadvertently shifted from diets containing ~12% fat to ~6% fat just prior to *C. jejuni *infection for the first passage. This error was not discovered until after the mice had been infected. A previous experiment that allowed a direct comparison of *C. jejuni *11168 infected C57BL/6 IL-10^-/- ^mice on the ~12% fat diet and adapted to the ~6% fat diet for at least two weeks prior to infection did not reveal a statistically significant difference in survival, gross pathology or histopathology (data not shown). Therefore, all subsequent passages included a similar dietary shift prior to inoculation in order to maintain constant dietary conditions in the mice across the four serial passages.

During the first three passages of the serial passage experiment, fecal *C. jejuni *populations were monitored by plating on *C. jejuni *selective medium; population sizes were scored on a semi-quantitative scale with ranks from 0 to 4 [40] (Figure 2). Briefly, colonization was scored as 0 if plates had no *C. jejuni *cfu, level 1 if plates had < 20 cfu, level 2 if plates had > 20 but < 200 cfu, level 3 if plates had > 200 cfu, and level 4 if plates were covered with a lawn of *C. jejuni*. Two-way ANOVA was performed on the ranked colonization data from the first three passages with the Holm-Šidák test for post hoc comparisons. For all strains except D0835, ranked population sizes varied with the day of sampling (P = 0.006 for strain 11168, 0.004 for strain D2586, 0.028 for strain D2600, and 0.009 for strain NW). In the four strains where significant differences were found, populations at the time of necropsy in almost all passages were larger than those on days 3 or 4 and sometimes larger than those on days 9 or 10. For strain 11168, population sizes on day 3 or 4 were significantly different from those both on day 9 or 10 and at the time of necropsy (P_corrected _= 0.01 and 0.02, respectively); population sizes on day 9 or 10 were not significantly different from those at the time of necropsy. Furthermore, significant differences in fecal population sizes between passages were found for strains 11168, D2600, and NW. For strain 11168, the comparison between passages was significant for the comparison of passage 1 to both passages 2 and 3 (P_corrected _= 6.8 × 10^-7 ^and 6.0 × 10^-8^, respectively) and for the comparison of passages 2 and 3 (P_corrected _= 1.2 × 10^-3^). For strains D2600 and NW, only the comparison between passages 1 and 3 was significant (P_corrected _= 7.4 × 10^-4 ^and 0.017, respectively).

The fraction of mice harboring *C. jejuni *in the jejunum also increased over the serial passage experiments for strains 11168, D0835, and D2600 (Additional file 1, Table S1). For strains 11168 and D0835, this fraction increased from 60% and 40%, respectively, to 100% after one passage in mice; for strain D2600, this fraction increased from 60% to 100% between the third and final passages. The changes in these proportions were significant by Fisher's exact test (P = 0.033 for strain 11168; P = 0.004 for strain D0835; P = 0.031 for strain D2600). In previous experiments, the jejunum was colonized in 30–60% of mice infected for 28–35 days with unpassaged *C. jejuni *11168 [40]. At the time of necropsy, levels of *C. jejuni *colonization in the cecum, the site where *C. jejuni *populations are highest and most consistent, were estimated on a semi-quantitative scale [40] and were similar for all five colonizing strains in all passages (data not shown).

**Figure 4:**
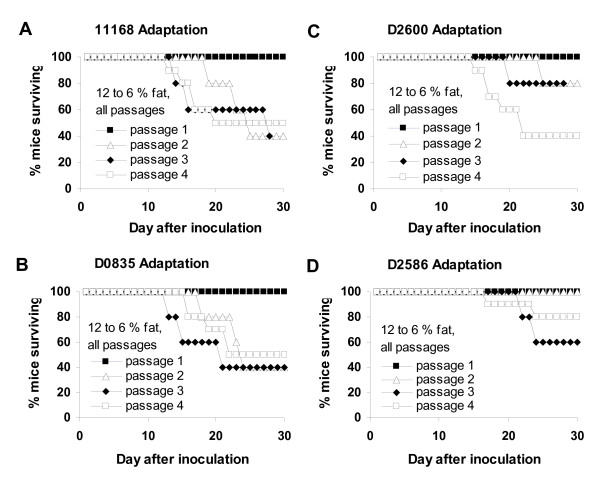
**Decrease in mouse survival in four passages during adaptation by serial passage (experiment 2)**. Panel A, *C. jejuni *11168; panel B, *C. jejuni *D0835; panel C, *C. jejuni *D2600; panel D, *C. jejuni *D2586. No control mice or mice infected with strain NW required early euthanasia (data not shown). All mice in all passages experienced a dietary shift from an ~12% fat diet to an ~6% fat diet 3 to 5 days prior to inoculation with *C. jejuni*. Passages 1, 2, and 3 had five infected mice each for each strain; passage four had 10 infected mice. Passage 1 had four sham inoculated control mice; passages 2 and 3 had five control mice each; passage four had 10 control mice.

In the first passage, all mice inoculated with all *C. jejuni *strains survived through the entire 30 days of the experiment. In the second passage, some mice inoculated with strains 11168, D0835, and D2600 required early euthanasia due to severe clinical disease (Figure 4). (For details of clinical scoring protocol, see Michigan State University (MSU) Microbiology Research Unit Food and Waterborne Diseases Integrated Research Network-sponsored Animal Model Phenome Database website http://www.shigatox.net/cgi-bin/mru/mi004). In the third passage, some mice inoculated with these strains and with strain D2586 required early euthanasia. In addition, the time between inoculation and the development of severe clinical disease requiring euthanasia decreased steadily over the second and third passages for strains 11168, D0835, and D2600. In all passages, all mice inoculated with strain NW survived for the full duration of the experiment (data not shown). Kaplan Meier log-rank survival analysis was conducted on the data for each strain from the four passages, although the number of animals (25) in each data set was low. Results were significant for strain D2600 (P = 0.028) but not for strains 11168, D2586, or D0835 (P = 0.264, 0.270, and 0.201, respectively). No mice infected with strain NW required early euthanasia.

**Figure 5:**
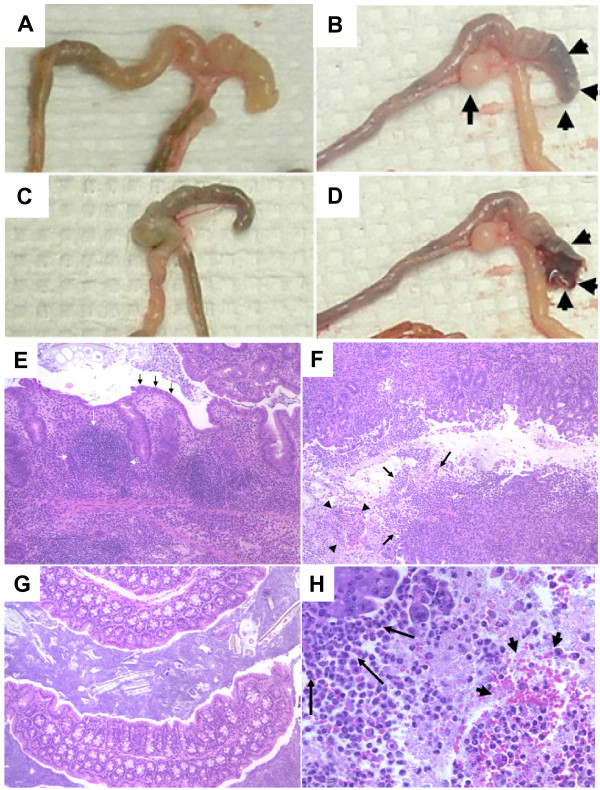
**Changes in gross and histopathology caused by *C. jejuni *strains during serial passage (experiment 2)**. C57BL/6 IL-10^-/- ^mice develop typhlocolitis with either "watery" contents (primary challenge) or "bloody" contents (after adaptation) following oral inoculation with *C. jejuni*. Panels A-D show images of gross pathology; panels E-H show images of histopathology from the same mice. Panel A shows thickened cecal and colon section with watery contents in a *C. jejuni *infected mouse 30 days after a primary challenge with strain 11168. Panels B and D show thickened cecal and colon section with bloody contents from a *C. jejuni *infected mouse 30 days after challenge with adapted strain 11168. Arrow indicates greatly enlarged ileocecocolic lymph node and arrowheads point to cecal tip with dark contents. In D cecal tip is opened to expose the frank blood (arrowheads). Panel C shows the cecum and colon of a normal sham inoculated control mouse. Panels E-H show histopathology from the same mice (E-G images taken at 10× magnification, H image taken at 40× magnification). Panel E shows mucosa of colon from the *C. jejuni *infected mouse with watery colon contents of Panel A. Note hyperplasia, intense mononuclear cell infiltration (white arrows) and slight neutrophilic exudates. Black arrows indicate the presence of intact epithelium. Panel F shows mucosa of colon from *C. jejuni *infected mouse with bloody colon contents from Panels B and D. This section also has intense cellular infiltrates, but it also has loss of epithelial layer (arrows) and blood and neutrophils present in exudates (arrowheads). Panel H shows a close-up of area in Panel F indicated with arrows. Long arrows point to sloughed villus tip epithelium. Arrowheads point to exudates with visible red blood cells and neutrophils. Panel G shows the colon mucosa of a normal sham inoculated control mouse for comparison.

**Figure 6:**
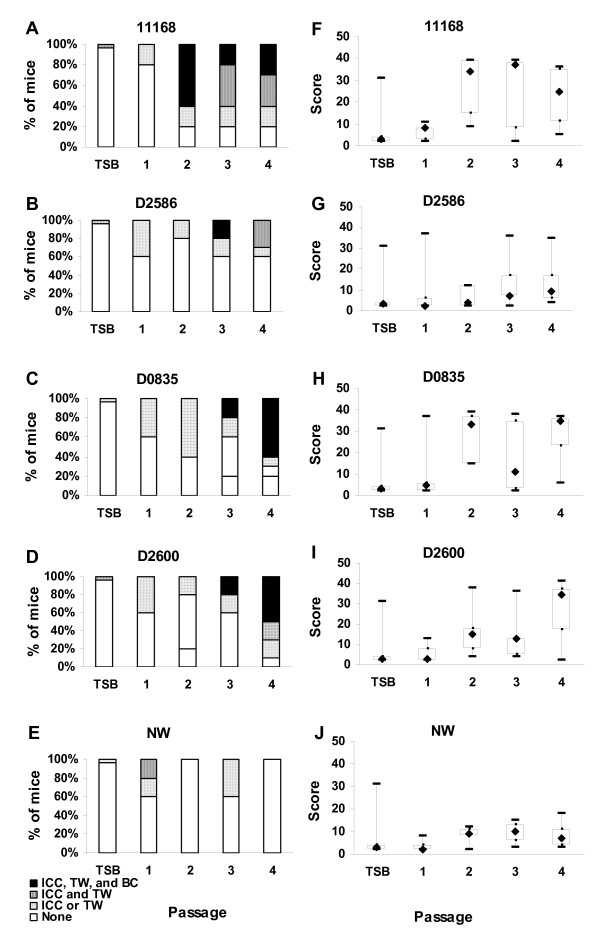
**Changes in gross and histopathology caused by *C. jejuni *strains during serial passage (experiment 2)**. Panels A-E, gross pathology; panels F-H, histopathology. In panels F-H, boxes enclose the central 50% of the scores; whiskers indicate the maximum and minimum scores; diamonds indicate the median score. All mice in all passages experienced a dietary shift from an ~12% fat diet to an ~6% fat diet 3 to 5 days prior to inoculation with *C. jejuni*. Passages 1, 2, and 3 had five infected mice each for each strain; passage four had 10 infected mice. Passage 1 had four sham inoculated control mice; passages 2 and 3 had five control mice each; passage four had 10 control mice. ICC, enlarged ileocecocolic lymph node; TW, thickened colon wall; BC, bloody contents in GI tract; TSB; sham inoculated control mice.

The fraction of mice exhibiting gross pathology in the gastrointestinal tract on necropsy (enlarged ileocecocolic lymph nodes, thickened and/or fluid-filled colon, bloody contents in lumen) increased during serial passage of three of the five strains. The increased occurrence of bloody contents in the GI tract lumen was a significant change from our observations in previous experiments (Figure 5). The severity of gross pathology, particularly the fraction of mice exhibiting bloody contents in the intestinal lumen (black sections of bars), increased in passaged strains 11168, D0835, and D2600 but not in passaged strains D2586 or NW (Figure 6A–E). In previous experiments, one of 82 *C. jejuni *11168 infected C57BL/6 IL-10^-/- ^mice had bloody contents in the intestinal lumen (1.2%), whereas in the second and subsequent passages in this experiment, 20 of 99 (20.2%) mice infected with passaged strains had this pathology. The single control mouse (1 of 29) having gross pathology and a high histopathology score tested negative for *C. jejuni *by both culture and PCR; it was thus a case of spontaneous colitis, which sometimes occurs in IL-10-deficient mice [45-48]. None of the 19 uninfected C57BL/6 IL-10^-/- ^mice with spontaneous colitis that we have observed in either our breeding colony or in experiments have exhibited bloody contents in the gut lumen. For each passaged *C. jejun*i strain, Kruskal Wallis ANOVA was performed to determine whether differences in the level of gross pathology in mice from the four different passages of that strain were statistically significant; results were significant for strain D2600 (P = 0.047) but not for strains 11168, D2586, or D0835 (P = 0.099, 0.859, and 0.221, respectively).

Median histopathology scores increased during serial passage of strains 11168, D0835, and D2600 (Figure 6F–J) but not strains D2586 and NW. This increase occurred after the first passage in strains 11168 and D0835 and after the third passage in strain D2600. The median histopathology score rose to over 30 in mice infected with strains 11168, D0835, and D2600; in previous experiments, the median histopathology score for mice infected with non-mouse-adapted *C. jejuni *11168 ranged from 9 to 19 [40]. Strain D2586 produced high histology scores in a few mice in the first, third and fourth passages, but the median score did not rise above 9. For each passaged *C. jejun*i strain, Kruskal Wallis ANOVA on ranks was performed to determine whether differences in the level of gross pathology in mice from the four different passages of that strain were statistically significant; results were significant for strain D2600 (P = 0.044) but not for strains 11168, D2586, D0835, or NW (P = 0.051, 0.827, 0.130, and 0.251, respectively). When post hoc multiple comparisons on the data for strain D2600 were done using the Holm-Šidák procedure, the result was significant for the comparison of histopathology scores of mice in passage 1 compared to the scores of mice in passage 4 (P_corrected _= 0.011).

Histopathology scores were also analyzed using the Mantel test for trends with correction for continuity [49]; for this test, data were cast in a two-way table for each *C. jejuni *strain according to the number of the serial passage of the strain and the number of animals exhibiting lesions of grades 0 and 1 combined (scores ≥ 0 and ≤ 19) compared to the number of animals exhibiting lesions of grade 2 (scores ≥ 20). The choice to divide the data in this way for this analysis was made because of the marked increase noted above in the median histopathology scores of *C. jejuni *11168 infected mice: from grade 1 in previous experiments to grade 2 after serial passage. The tests for trends were statistically significant for strains 11168 (χ^2 ^= 16.47; d.f. = 1; 0.00001 < P < 0.0001), D0835 (χ^2 ^= 18.25; d.f. = 1; 0.00001 < P < 0.0001), and D2600 (χ^2 ^= 16.90; d.f. = 1; 0.00001 < P < 0.0001). The test was not significant for strain D2586 (χ^2 ^= 2.14; d.f. = 1; 0.14 < P < 0. 15) and could not be conducted for strain NW since there were no NW-infected mice having histopathology scores in grade 2.

### DNA:DNA microarrray comparison of *C. jejuni *strains 11168 and NW (experiment 3) revealed differences between the strains

Because strain NW was able to colonize C57BL/6 IL-10^-/- ^mice but did not cause severe enteritis in the initial infection and did not evolve to a higher level of pathogenicity during repeated passages, we elected to examine its genetic content more closely by comparing it to the highly pathogenic strain 11168 using an in-house full open reading frame (ORF) microarray with coverage of 95% of the *C. jejuni *11168 genome [50]. The microarray was constructed using PCR products synthesized using primers for sequence-validated ORFs developed by Parrish *et al*. [51] and genomic DNA from strain 11168 (See NCBIGEO series number GSE13794 for a description of chip manufacture.) We hypothesized that known virulence determinants would be among the genes present in strain 11168 but absent from strain NW.

Sixty-nine *C. jejuni *11168 ORFs were identified as possibly absent in strain NW by Genomotyping (GACK) analysis of microarray data [52]. Fifty-four of the 69 ORFs were confirmed to be absent or strongly divergent by PCR assay (Additional file 1, Table S2); PCR products of the appropriate size were obtained for thirteen of the remaining ORFs. Many of the ORFs missing in strain NW belong to complex loci encoding surface structures known both to be involved in *C. jejuni *pathogenesis and to be highly variable in gene content (flagellin, 8 ORFs; capsule, 11 ORFs; LOS, 1 ORF (*gmh*A); [53]). Nine additional ORFs may encode membrane proteins; three may encode DNA restriction and modification proteins. Four periplasmic proteins were absent or strongly divergent in strain NW, along with seven ORFs having other known or putative functions and 11 ORFs encoding hypothetical proteins for which no function could be suggested [53].

For two ORFs, Cj 0987c (putative integral membrane protein) and Cj0874c (possible cytochrome c protein), strain NW DNA yielded PCR products smaller than those produced from strain 11168 DNA. Sequencing of the PCR products from strain NW showed that Cj0987c had a 649 bp deletion (nucleotides 121–770 of Cj0987c from strain 11168) compared to strain 11168. ORF Cj0874c in strain NW had a 182 bp deletion (nucleotides 212–393 of Cj0874c from strain 11168) compared to strain 11168. GenBank accession numbers for these sequences are FJ361181 and FJ361182.

### Early course of infection was not altered by serial passage of *C. jejuni *11168 (experiment 4; short term infection)

The observation that fecal *C. jejuni *11168 population sizes increased during the course of infection, the significant increase in fecal population sizes of this strain with passage, and the earlier onset of severe enteritis that occurred during passage of *C. jejuni *strain 11168 led us to hypothesize that serial passage might have selected for variants that were more proficient in growth in the host immediately after infection and/or in early initiation of the disease process. Therefore, we infected mice with passaged and unpassaged *C. jejuni *11168 and compared levels of colonization, gross pathology, and histopathology in the two groups 48 hours after infection. The results did not support the hypothesis. Mice infected with the two strains did not differ in colonization at different sites in the GI tract (Additional file 1, Table S1) or in colonizing population sizes (data not shown). Four of ten mice infected with unpassaged and four of ten mice infected with passaged *C. jejuni *11168 exhibited slightly enlarged lymph nodes; all mice had minimal histopathology scores between 2 and 5 (grade 0; data not shown).

### Adaptive humoral immune responses were not consistently affected by passage of *C. jejuni *strains (experiment 2; serial passage experiment)

**Figure 7:**
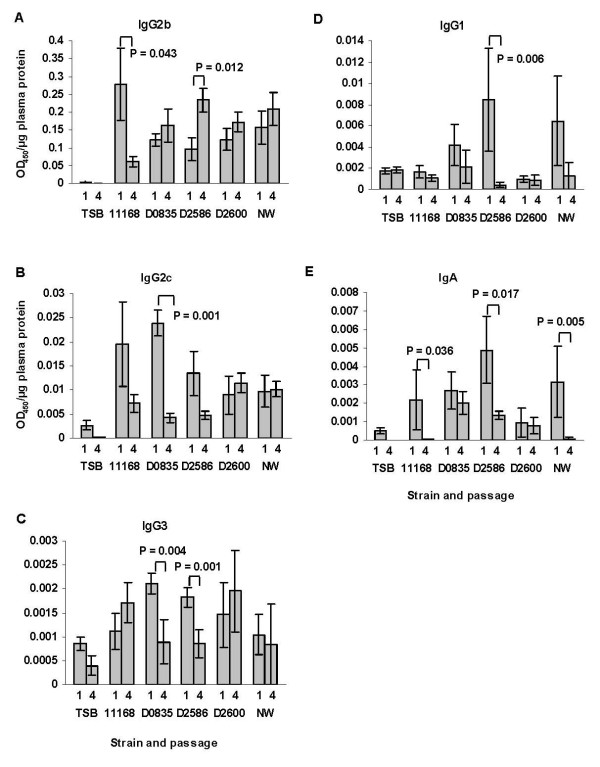
**Plasma anti-*C. jejuni *antibody levels in mice infected with different *C. jejuni *strains (experiment 2)**. Panel A, IgG2b; Panel B, IgG2c; panel C, IgG3; panel D, IgG1; and panel E, IgA. Antibody levels for the first and fourth passage are shown. Bars represent the average OD_450_/μg plasma protein of mice in passages 1 and 4; whiskers indicate standard error. P values of statistically significant differences between antibody level in passage 1 compared to passage 4 for individual strains are shown on the graph. TSB, sham inoculated control mice.

ELISA tests were performed to characterize the adaptive immune responses of the mice to the evolving strains (Figure 7A–E); the antigen for all of these assays was prepared from non-adapted (unpassaged) *C. jejuni *11168. The response of C57BL/6 IL-10^-/- ^mice to *C. jejuni *was previously shown to be dominated by Th1-associated antibodies, predominantly IgG2b [40]; the same result was obtained for the other colonizing strains. There were a few cases in which anti-*C. jejuni *IgG subclass antibody titers were significantly decreased in the serum of mice infected with the passaged strain in the last passage compared to the initial passage. Anti-*C. jejuni *IgA titers were significantly lower in mice infected with three of the five passaged strains (11168, D2586, and NW) in the last passage compared to the initial passage; however, of those three strains, only *C. jejuni *11168 increased in pathogenicity during passage. Also, anti-*C. jejuni *11168 specific IgA responses of the mice challenged with non-colonizing strains 33560 and D0121 were high and low, respectively, suggesting that these responses did not correlate with clearance of the organism from the GI tract. Although mucosal IgA responses were not measured, these may better correlate with clearance of a particular *C. jejuni *strain from the GI tract.

### Percentage of fat in and/or fatty acid composition of diet influenced disease expression during infection with unpassaged *C. jejuni *11168 (experiment 2, serial passage experiment; and experiment 5, diet comparison)

The two diets fed to the mice in these studies differed principally in fat composition (an ~12% minimum for the breeder diet and an ~6% minimum for the NIH-31 formula maintenance diet) and linoleic acid content (0.62% for the ~12% fat diet and 2.55% for the ~6% fat diet), although a number of other constituents were also different. Both diets contained wheat, corn, and soybean meal. The ~12% fat diet also contained porcine fat, whey, casein, lecithin, and soybean meal and hulls, whereas the ~6% fat diet contained oats, wheat middlings, fish meal, soybean oil, alfalfa meal, and corn gluten meal. Results from a previous unrelated experiment did not show any significant differences in survival, gross pathology, or histopathology between groups of *C. jejuni *11168 infected C57BL/6 IL-10^-/- ^mice kept on the ~12% fat diet and mice kept on the ~6% fat diet throughout the experiment (data not shown; [54]). However, since mice in that previous experiment were shifted from the ~12% fat diet to the ~6% fat diet at least two weeks prior to inoculation, the dietary conditions were not exactly comparable to those experienced by mice undergoing the dietary transition just prior to inoculation. Therefore we compared mice infected with non-adapted *C. jejuni *11168 on the ~12% fat diet and mice experiencing the transition from the ~12% fat diet to the ~6% fat diet in conjunction with the final phase of the serial passage experiment.

**Figure 8:**
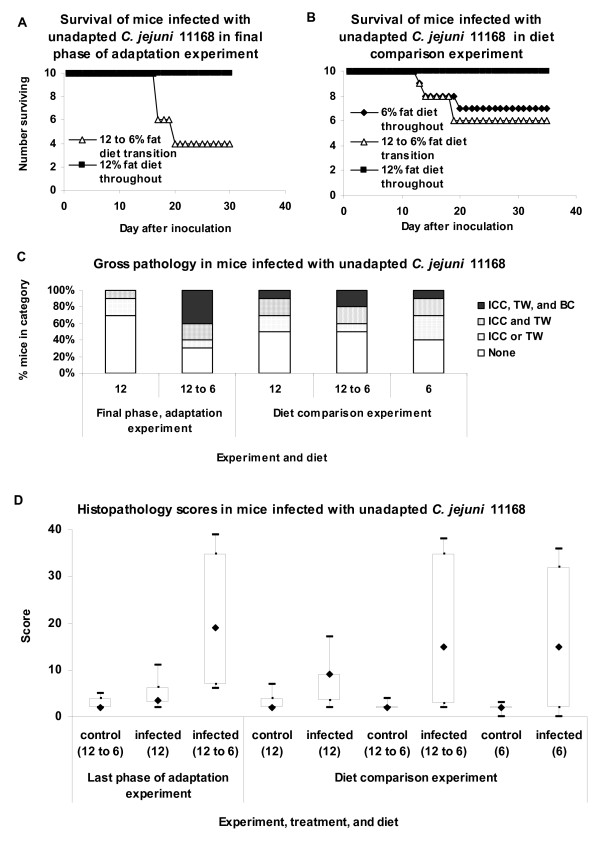
**Survival, gross and histopathology in mice on different dietary regimes (experiments 2 and 5)**. Results from two comparisons are shown. One comparison of infected mice on the ~12% fat diet with infected and control mice that experienced a dietary shift from a ~12% fat diet to an ~6% fat diet 3 to 5 days prior to inoculation with *C. jejuni *was conducted concomitantly with the final phase of experiment 2 (serial passage experiment). In experiment 5 (diet comparison), the balanced design included control and infected mice kept on the 12% fat diet throughout the experiment, kept on the 6% fat diet throughout the experiment, or subjected to a transition from the 12% fat diet to the 6% fat diet just prior to inoculation. No sham-inoculated control mice (TSB, tryptose soy broth) required early euthanasia or showed gross pathological changes on necropsy; data are not shown. In panel D, boxes enclose the central 50% of the scores; whiskers indicate the maximum and minimum scores; diamonds indicate the median score. ICC, enlarged ileocecocolic lymph node; TW, thickened colon wall; BC, bloody contents in GI tract; TSB; sham inoculated control mice.

In the diet comparison conducted in the final phase of experiment 2 (serial passage experiment), six of ten mice infected with non-adapted *C. jejuni *11168 that experienced the transition from the ~12% fat diet to the ~6% fat diet required early euthanasia due to disease but no mice infected with non-adapted *C. jejuni *11168 and kept on the ~12% fat diet throughout the experiment did so (Figure 8A). Kaplan Meier log rank survival analysis showed that the difference in survival was statistically significant (P ≤ 0.001). Post hoc comparisons were significant for comparisons of (1) infected mice on the two diets and (2) control mice experiencing the transition from the 12% fat diet to the 6% fat diet to infected mice experiencing the transition from the ~12% fat diet to the ~6% fat diet at the time of inoculation (P_corrected _= 0.014 for both comparisons). In addition, in the diet comparison conducted in the final phase of experiment 2 (serial passage experiment), there were significant differences in gross pathology (P = 0.002 for Kruskal Wallis ANOVA; Figure 8C). However, while mice infected with non-adapted *C. jejuni *11168 that experienced the transition from the ~12% fat diet to the ~6% fat diet were significantly different in gross pathology from controls experiencing the dietary transition (P_corrected _= 0.009), the post hoc comparisons of (1) infected mice on the ~12% fat diet to control mice and (2) infected mice on the two diets were not significant (P_corrected _= 0.087 and 0.105, respectively). Finally, there were also significant differences in histopathology (P ≤ 0.001 for Kruskal Wallis ANOVA; Figure 8D) in the diet comparison conducted in the final phase of experiment 2 (serial passage experiment). In post hoc comparisons, infected mice experiencing the transition from the ~12% fat diet to the ~6% fat diet at the time of inoculation experienced significantly greater histopathology (P_corrected _= 0.033) than control mice experiencing the dietary transition. However, post hoc comparisons of infected mice on the ~12% fat diet to (1) infected mice experiencing the dietary transition and (2) control mice experiencing the dietary transition were not significant (P_corrected _= 0.057 and 1.0, respectively).

Since different outcomes were observed in two experiments, we conducted another experiment (experiment 5, diet comparison) with a balanced design that allowed a full comparison of mice infected with non-adapted *C. jejuni *11168 on three diet regimes (~12% fat diet throughout, ~6% fat diet throughout, and transition from the ~12% fat diet to the ~6% fat diet just prior to inoculation) and control mice on each of the three diet regimes. Three infected mice kept on the ~6% fat diet throughout required early euthanasia, as did four mice that experienced the transition from the ~12% fat diet to the ~6% fat diet (Figure 8B). No mice kept on the ~12% fat diet throughout required early euthanasia. When the survival curves of the three groups of infected mice were compared, the Kaplan Meier statistic was not significant (P = 0.105).

In experiment 5 (diet comparison), levels of gross pathology in infected mice were similar in all groups of mice (Figure 8C); no control mice exhibited gross pathology. When gross pathology scores of the six groups of mice were analyzed using two-way ANOVA on ranked data, differences among the groups due to infection status were significant (P_controls vs infected _= 6.11 × 10^-24^), but there was no statistically significant difference due to diet (P = 0.956), nor was there a statistically significant interaction between infection status and diet (P = 0.956).

Histopathology scores were elevated both in infected mice kept on the ~6% fat diet throughout and in infected mice experiencing the transition from the ~12% fat diet to the ~6% fat diet (Figure 8D). When histopathology scores of the six groups of mice were analyzed using two-way ANOVA on ranked data, differences among the groups due to infection status were significant (P_controls vs infected _= 2.33 × 10^-6^), but there was no statistically significant difference due to diet (P = 0.553). Nor was there a statistically significant interaction between infection status and diet (P = 0.611).

**Figure 9:**
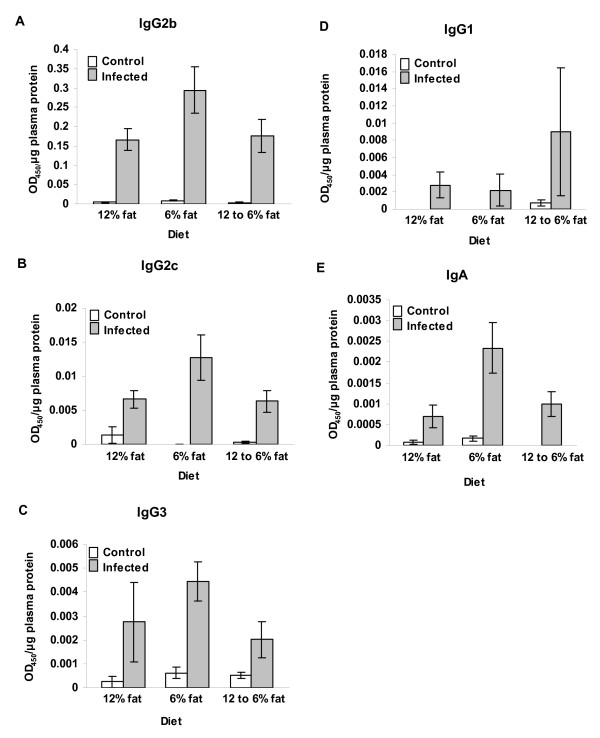
**Plasma anti-*C. jejuni *antibody levels in mice on different dietary regimes (experiment 5)**. A comparison with a balanced design included infected and control mice kept on the ~12% fat diet, kept on the ~6% fat diet, or experiencing a transition from the ~12% fat diet to the ~6% fat diet just prior to inoculation. Panel A, IgG2b; Panel B, IgG2c; panel C, IgG3; panel D, IgG1; and panel E, IgA. Bars represent the average OD_450_/μg plasma protein of 10 mice; whiskers indicate standard error. P values of statistically significant differences between antibody levels are shown on the graph. TSB, sham inoculated control mice.

Humoral immune responses to *C. jejuni *infection of mice on the different dietary regimes in experiment 5 (diet comparison) are shown in Figure 9. When two-way ANOVA was conducted on these data, the effect of infection status (infected *vs *controls) was significant for plasma levels of anti-*C. jejuni *IgG2b, IgG2c, IgG3, and IgA (P = 1.68 × 10^-10^, 8.93 × 10^-7^, 8.57 × 10^-7^, and 5.34 × 10^-6^, respectively) but not for IgG1 (P = 0.109). There was no statistically significant effect of diet on levels of anti-*C. jejuni *IgG2b, IgG2c, IgG3, or IgG1 (P = 0.114, 0.203, 0.204, and 0.477, respectively). There was no statistically significant interaction between diet and infection status for anti-*C. jejuni *IgG2b, IgG2c, IgG3, or IgG1 (P = 0.202, 0.075, 0.076, and 0.620, respectively). However, for plasma anti-*C. jejuni *IgA, there was a statistically significant effect of diet (P = 0.012) as well as a significant interaction between diet and infection status (P = 0.035). Plasma IgA levels were significantly different in mice on the ~6% fat diet compared to mice on the ~12% fat diet (P_corrected _= 0.019) and in mice on the ~6% fat diet compared to mice experiencing the transition between the two diets at the time of inoculation (P_corrected _= 0.032). Plasma IgA levels in mice experiencing the dietary transition were not significantly different from those of mice on ~12% fat diet (P = 0.695).

## Discussion

### Outcome of infection was influenced by genetic differences between *C. jejuni *strains

MLST analysis of over 3000 strains has provided a comprehensive picture of genetic relationships among *C. jejuni *strains (*Campylobacter jejuni *Multi Locus Sequence Typing website [7]). The seven strains used in this study represent six different MLST sequence types with varying degrees of genetic relatedness among them. Genetic relationships of the seven strains derived from pathogenicity gene RFLP analysis were roughly consistent with those derived from MLST data. However, only a small number of strains were examined, and this congruence may not be substantiated when more strains are examined. Disease outcomes were consistent with genetic relationships in that the two strains that failed to colonize C57BL/6 IL-10^-/- ^mice clustered at a distance from the colonizing strains in both MLST and pathogenicity gene RFLP analyses. However, strains 11168 and D2586 were identical in the RFLP analysis of virulence loci, and while strain D2586 was able to colonize the mice at high levels and cause some disease, it did not increase in pathogenicity in the course of four serial passages. (It is of course possible that strains D2586 and NW might increase in pathogenicity if passages were continued.) These results support the hypothesis that there are genetic differences between *C. jejuni *strains that can influence the ability of a given strain to interact with the host. Furthermore, the observation that there are differences between *C. jejuni *strains in the ability to produce enteritis in C57BL/6 IL-10^-/- ^mice shows that the development of severe disease cannot be solely attributed to the immune alterations of the mice.

All strains used in these experiments possessed twelve known and putative virulence loci for which presence/absence polymorphisms have been reported in the literature. Two putative *C. jejuni *virulence determinants, *iam *and *wla*N, which were absent in one or more of the strains used in this study, are probably not required for pathogenicity in mice. The *iam *marker was first identified as a DNA fragment obtained in a random amplified fragment polymorphism analysis; this fragment was epidemiologically linked to *C. jejuni *associated disease [19]. However, another epidemiological study did not reveal a high prevalence of this marker in *C. jejuni *strains from diseased patients [55], and it has recently been shown that the *iam *gene makes little or no contribution to invasion of cultured INT407 cells by *C. jejuni *strains possessing it [56]. Our results are in accordance with these findings in that strains D0835 and D2600, which were able to cause enteritis in C57BL/6 IL-10^-/- ^mice, were *iam*^-^. Both the *cgt*B and the *wla*N genes encode β-1,3- galactosyltransferases thought to be involved in synthesis of the *C. jejuni *outer membrane lipo-oligosaccharide. All strains in this study possessed *cgt*B, and all except D2600 possessed *wla*N. Muller *et al*. [57,58] studied the associations of these genes with the ability to invade Caco-2 cells in culture and to colonize chickens; their results suggest that possession of one or both genes is associated with the ability to invade eukaryotic cells and to colonize the chicken GI tract, but one strain that lacked both loci was fully invasive.

### Adaptation to the host by serial passage altered the outcome of infection for three of five *C. jejuni *strains

Three of five *C. jejuni *strains (11168, D0835, and D2600) became more virulent during serial passage in mice as shown by increased colonization of the jejunum, decreased time to develop clinical disease, and increased levels of both gross pathology (particularly increased incidence of bloody diarrhea) and histopathology. Fecal population sizes of two of the three strains that became more virulent increased during serial passage. The change toward increased pathogenicity in the three evolving strains occurred after one passage in two strains and after three passages in one strain. This observation suggests that the strain that increased in pathogenicity only after three passages may have had to undergo more extensive genetic change than the other two strains. An increase in pathogenicity is consistent both with a large body of theoretical work and with previous experimental studies of pathogenicity evolution; in this case, since the mice were individually housed, virulence trade-offs with transmission dynamics between hosts would not be expected to occur. Since all mice in all passages of the serial passage experiment experienced the same dietary conditions (transition from the ~12% fat breeder diet to the ~6% fat NIH-31 formula maintenance diet), differences in the behavior of a *C. jejuni *strain in different passages cannot be attributed to differences in diet, particularly for strain D2600, which did not show increased colonization of the jejunum or marked increases in pathology until after the third passage.

Two *C. jejuni *strains, D2586 and NW, did not increase in pathogenicity during four serial passages. Although we cannot rule out the possibility that continued passage might have produced an increase in pathogenicity in these strains, this result shows that the initial genetic complements of the two strains affected their ability to respond to the selection pressure imposed by the novel host environment of the mouse GI tract.

Microarray comparison of the gene content of strain NW to that of strain 11168 revealed that strain NW did not possess a detectable homologue of *C. jejuni gmh*A, a gene involved in LOS/LPS synthesis encoding sedoheptulose-7-phosphate isomerase. This enzyme is essential for production of the heptose residues of the inner core of the LOS/LPS structure. In *Haemophilus ducreyi*, inactivation of the *gmh*A gene has been shown to result in a truncated LOS and to reduce the ability of the organism to produce skin lesions in rabbits [59]. In addition, the ability of *Salmonella enterica *to kill *Caenorhabditis elegans *was impaired by insertional inactivation of the *gmh*A gene [60]. Mutation of another *C. jejuni *gene involved in synthesis of the LOS inner core, *waa*C, markedly impaired the ability of *C. jejuni *81–176 to invade the intestinal cell line INT407 *in vitro *[61]. Strain NW was also missing a number of *C. jejuni *11168 genes in complex loci involved in capsule synthesis and O-linked glycosylation of the flagellin protein. Extensive variation in these loci has been reported in other microarray comparisons of *C. jejuni *strains [12]. Both flagella and capsule have been reported to affect virulence in *C. jejuni *[18,24]. The reason for the inability of strain D2586 to increase in virulence is not known, but a similar approach could be taken to examine gene content in comparison to strain 11168.

The degree and complexity of the phenotypic changes we observed – increased fecal population sizes, increased colonization of the jejunum, decreased time to develop severe disease, shift from watery to bloody diarrhea – suggest that the three evolving strains underwent genetic change at multiple loci, including loci that influence growth and loci that influence interaction with and damage to host tissues. We have no information on any specific genetic changes that led to these phenotypic changes at the present time; further studies on these strains will utilize gene expression microarrays to focus on the hypothesis that the changes in pathogenicity are due to changes in gene expression levels or patterns; experimental infection of C57BL/6 IL-10^-/- ^mice with *C. jejuni *11168 derivatives containing targeted gene knockouts will be used to determine whether corresponding genes contribute to virulence in *C. jejuni *11168.

### Outcome of *C. jejuni *infection and host immune response were influenced by diet

Results from two of three trials (the previous experiment with mice kept on an ~12% fat diet and an ~6% fat diet throughout the experiment and the full, balanced design comparison (experiment 5, diet comparison) of the effect of diet on the outcome of *C. jejuni *infection) did not indicate that there was an effect of diet on survival, gross pathology, or histopathology in mice infected with unpassaged *C. jejuni *11168. On the other hand, results from the diet comparison conducted in the final phase of experiment 2 (serial passage experiment) did indicate such an effect. In addition, there was a significant effect of diet on plasma IgA levels in the full, balanced design experiment (experiment 5, diet comparison). The latter result indicates that some constituent of the diet, possibly the amount and/or isomeric composition of linoleic acid, could have affected the immune responses of the mice. Linoleic acid, which is known to affect the immune response, was present at ~0.6% in the 12% fat diet and ~2.6% in the 6% fat diet.

The latter results – taken together with the considerable body of literature implicating specific isomeric forms of various dietary lipids, including linoleic acid, as immune system modulators [62,63] at levels comparable to those in the mouse diets we used [64] and with findings that different dietary lipids can affect the process of infection with *Listeria monocytogenes *[64-66] – suggest that dietary factors, possibly lipid composition, may affect the outcome of *C. jejuni *infection in C57BL/6 IL-10^-/- ^mice. The manufacturer of the mouse chow we used does not report the isomeric composition of the total linoleic acid, which is derived from fish meal, soybean, and grains, and which might be expected to vary from batch to batch. It would therefore be difficult or impossible to determine retrospectively whether the chow fed to the mice in the three experiments was different in composition. Finally, it is also possible that the differing constituents of the two diets influenced either the host immune system or the indigenous intestinal microbiota or both in such a way as to affect the pattern or level of disease expression due to *C. jejuni *infection. Experiments using mice fed defined diets would be required to explore these effects.

There was no indication from the ELISA results that antibody responses were protective in C57BL/6 IL-10^-/- ^mice against infection with any of the tested strains of *C. jejuni *used for challenge. The majority of infected mice produced robust Th1 associated IgG2b responses to all *C. jejuni *strains tested; this response was associated with disease except in strains D2586 and NW. Infected non-colonized mice did not produce strong IgG2b responses. Also, other antibody responses in plasma of all infected mice were low. However, there were some significant differences between the first and last passage in levels of anti-*C. jejuni *11168 IgG2b antibodies detected by ELISA in mice challenged with various *C. jejuni *strains. We suspect that these differences reflected changing surface antigenic structures of the *C. jejuni *strains during repeated passage that made them more or less similar to antigen from the unadapted 11168 strain used to coat the ELISA plates. Thus, strain 11168 changed over passage so that mice in the last passage had significantly less anti-non-adapted 11168 IgG2b antibodies than mice in the first passage. This speculation would have to be followed up with further experiments to test this hypothesis. In contrast, mice challenged with strain D2586 in the fourth passage produced IgG2b antibodies that recognized non-adapted strain 11168 ELISA antigens better than mice in the first passage experiment. In addition, there was no correlation between any immunoglobulin isotype and colonization (rank abundance) of any *C. jejuni *strain at day 30 after infection. This result is in contrast to those of Fox *et al*. where C57BL129 mice infected with *C. jejuni *81–176 cleared their infections 60 days after challenge and clearance was correlated with lower Th1 associated IgG2a responses [67]. Furthermore, in our dataset it was interesting that in the first round of *C. jejuni *challenges the highest (and most variable) Th2 associated IgG1 responses were seen in mice receiving the colonizing strains that caused little or no disease or lesions. A similar pattern was observed in IgA responses. In mice in groups receiving the nonpathogenic *C. jejuni *strains NW and D2586, continued adaptation of the strain elicited significantly less IgA and, in the case of D2586, less IgG1. Taken together these results suggest that there is variability in ability of *C. jejuni *strains to elicit Th2 associated immunoglobulins and that this variability is affected by adaptation to the host, although the impact of this change on colonization and disease status is not clear. Further work is needed to examine anti-*C. jejuni *strain specific IgA levels in the gastrointestinal tract where IgA exerts its main effect.

## Conclusion

The results reported here show that *C. jejuni *strains from humans, chickens, and cattle vary in their ability to colonize and cause enteritis in C57BL/6 IL-10^-/- ^mice. Furthermore, serial passage of *C. jejuni *strains in C57BL/6 IL-10^-/- ^mice as well as dietary factors increase disease expression in this mouse model. Thus, the C57BL/6 IL-10^-/- ^mouse model can be used to detect differences in pathogenicity of different *C. jejuni *strains and is suitable for screening clinical isolates from different human disease states or for screening *C. jejuni *strains carrying disrupted putative virulence genes. The ORFs identified here as present in *C. jejuni *strain 11168 and absent in strain NW will be disrupted and screened for their role in pathogenicity. Furthermore, the model offers the opportunity to dissect the complex interactions between host genetics, host immune responses, pathogen genetics, and environmental factors such as diet and the indigenous microbiota that ultimately determine the course and outcome of infection. Such studies would clearly enhance investigations of *C. jejuni *virulence mechanisms and perhaps lead to improved options for prevention and treatment of this common disease.

## Methods

### Animals

All animal experiments were conducted according to NIH guidelines and were approved by the MSU All University Committee on Animal Use and Care. C57BL/6 IL-10^-/- ^mice (B6.129P2-IL10^*tm1Cgn*^/J) were originally obtained from the Jackson Laboratories (Bar Harbor, Maine); breeding mice were maintained and monitored in a specific-pathogen-free colony at MSU as previously described [40]. All mice used in these studies were produced in the on-campus breeding colony. Experiments were conducted in the University Research Containment Facility at MSU. Mice were maintained on two diets: irradiated mouse breeder diet 7904 (~12% fat content) and irradiated mouse NIH-31 formula maintenance diet 7913 (~6% fat content) (Harlan Teklad, Indianapolis, IN). Mice were weaned onto the ~12% fat diet at three weeks of age and then either kept on that diet, gradually shifted to the ~6% fat diet at least two weeks prior to inoculation with *C. jejuni *at 8 to 12 weeks of age or shifted abruptly to the ~6% fat diet just prior to inoculation at 8 to 12 weeks of age.

### *C. jejuni *strains

Details concerning the strains used appear in Table 1. Growth media and inoculum preparation were as previously described [40].

### Genetic methods

Total DNA was extracted from tissue and fecal samples using DNeasy Tissue Kit (Qiagen, Valencia, CA) and was assayed by species-specific PCR for the *C. jejuni gyr*A gene as previously described [40,44]. Pathogenicity gene complements of the *C. jejuni *strains were determined using published PCR assays cited in Table 2; the 9.6 kbp LOS fragment was generated using the Expand Long Template PCR System (Roche, Mannheim, Germany). Primers for *lux*S were generated using the web-based Primer3 program [68] http://jura.wi.mit.edu/rozen/papers/rozen-and-skaletsky-2000-primer3.pdf: GGTTGTCGCACGGGTTTTTA (forward) and GGCAATTTGTTTGGCTTCAT (reverse). Cycling conditions were 2.0 mM MgCl_2_, denaturation at 95°C for 1 min followed by 30 cycles of 94°C for 30 s, 49°C for 1 min, 72°C for 2 min, and final extension at 72°C, 10 min. RFLP analysis of virulence determinants was conducted as follows. PCR products for *fla*A, LOS, *cdt*ABC, *ceu*E, *pld*A, *cia*B, *dna*J, and *cgt*B were digested with *Dde*I, *Rsa*I, or *Hha*I to generate restriction fragment length polymorphism (RFLP) patterns. DNA extraction from bacterial cultures, restriction enzyme digestion, agarose gel electrophoresis, and ethidium bromide staining were performed using standard methods [69]. Stained gels were visualized and photographed using an Alpha Innotech UV transilluminator (Alpha Innotech, San Leandro, CA). Banding patterns were scored visually.

Multilocus sequence typing (MLST) of strain NW (GenBank accession numbers FJ361183 through FJ361189) was performed using genes, primer sets, and cycling conditions described at the *Campylobacter jejuni *Multi Locus Sequence Typing website http://pubmlst.org/campylobacter/ developed by Keith Jolley and Man-Suen Chan and sited at the University of Oxford [7]. DNA sequencing was performed at the MSU Genomics Technology Support Facility. Each PCR product was initially sequenced in both directions; additional sequencing was done as necessary to resolve discrepancies.

### DNA:DNA microarrray comparison of *C. jejuni *strains 11168 and NW

An in-house whole-open-reading frame (ORF) microarray for *C. jejuni *11168 (95% coverage) was developed using primers and clones described in Parrish et al. [51]. See NCBIGEO series number GSE13794 for a full description of chip manufacture. ORFs from pVir, *C. jejuni *strain 81–176 were also spotted on the chips. PCR products were generated from the cloned ORFs using the same primers and spotted in triplicate on Corning UltraGAP Superamine slides (Corning, Inc., Corning NY) using an Affymetrix GeneChip instrument at the MSU Research Technology Support Facility (RTSF).

Each strain was streaked from frozen stock on two tryptose soya agar plates containing 5% defibrinated sheeps' blood; plates were incubated for 48 hours at 37°C in 5% CO_2_. A single isolated colony from each plate was chosen and streaked onto another plate (biological replicates).

Growth from each of the second plates was harvested separately and genomic DNA was isolated using the CTAB procedure described in Ausubel et al. [69]. One μg DNA was sheared by sonication to 0.5–2.0 kbp and labeled with aminoallyl-dUTP using the BioPrime random priming DNA labeling kit^® ^(Invitrogen, Carlsbad, CA). Unreacted components were removed using a Qiagen PCR Purification kit^® ^(Qiagen, Valencia, CA). Aliquots of the purified aminoallyl-dU-containing DNA were then reacted with Cy5 or Cy3 dye (Amersham, Piscataway, NJ). Unreacted dye was removed using a Qiagen PCR Purification kit^®^. The two separate DNA extractions done for each strain were used in separate hybridizations (technical replicates). The experiment was repeated with the dyes reversed; thus a total of four chips were hybridized and compared for each strain. The spots for each gene are duplicated three times on each chip, for a total of 12 comparisons for each strain.

For hybridization, Ambion SlideHyb solution (Ambion, Austin, TX) was preheated to 54°C. The combined Cy3/Cy5 labeled DNA samples were resuspended in 4 μl 10 mM EDTA. The sample was then denatured at 95°C for 10 minutes. During this time the cover slip was washed in 95% ETOH, 0.1% SDS and sterile ddH_2_O. Cover slips were dried with filtered air. After denaturation of sample, 30 μl of prewarmed Ambion SlideHyb solution was added. The slides were centered on a warmed hybridization cassette and a cleaned cover slip was placed face down and centered over the spots. The denatured sample was then gently pipetted using capillary action to fill the area underneath the cover slip. Sixteen μl of ddH_2_O was added to the grooved edge of each hybridization chamber. The top of the hybridization chamber was then secured; the slides were placed on a rack in a 54°C water bath overnight. All steps were performed in the dark.

Post-hybridization washes were performed as follows. In the dark, the opened cassette was gently moved up and down in warmed 1 × SSC, 0.2% SDS until the cover slip fell off. The slide was placed on an orbital platform shaker at low speed for 4 minutes in the dark. The slide was transferred to 0.1 × SSC containing 0.2% SDS and incubated on the platform shaker at low speed for 4 min. The process was repeated twice with 0.1 × SSC. The slide was placed in a 50 ml conical tube covered with aluminum foil and centrifuged at 1000 rpm in a clinical centrifuge in a swinging bucket rotor for 5 min.

The slides were scanned in an Affymetrix 428 scanner and a "QuickScan" preformed to determine the quality and intensity of the slide; then the region of interest was defined and a full scan for Cy5 was done first, Cy5 being the more "fragile" of the two dyes. Each slide was scanned with three different gain levels to ensure equal intensity comparisons in later analyses. The process was repeated for Cy3. All scans were saved. Images were edited using GenPixPro3 (Molecular Devices, Sunnyvale, CA). *C. jejuni *11168 ORFs were identified as possibly absent in strain NW based on a relative fluorescence intensity of -0.5 for strain NW compared to strain 11168 for six of the twelve spots compared (see Statistical Methods section below).

To confirm the absence of ORFs meeting this criterion, DNA from strain NW and from *C. jejuni *11168 (positive control) was subjected to PCR assay using the primers described in Parrish et al. [51]. ORFs for which PCR product of the appropriate size was obtained for strain 11168 but for which no PCR product was obtained for strain NW were considered to be absent or strongly divergent in strain NW. To verify the identities of some ORFs, PCR products were partially sequenced at the MSU RTSF using the same primers used to generate the product on an ABI Prism^® ^3100 Genetic Analyzer. Each PCR product was sequenced in both directions.

### Experimental methods and designs

Full details of all experimental methods used in the murine model of *C. jejuni *infection are available in Mansfield et al. [40] and at the MSU Microbiology Research Unit Food and Waterborne Diseases Integrated Research Network-sponsored Animal Model Phenome Database website http://foodsafe.msu.edu/mru_web/MurineEntericDiseasesPhenomeDatabase.htm.

For adaptation by serial passage, five mice were inoculated with each *C. jejuni *strain in the first passage with inoculum prepared from frozen stock cultures as described [40]. In the initial passage, a fecal pellet collected from each mouse on days 3 or 4, 9 or 10, and 20 or 21 was suspended in tryptose soya broth (TSB) and streaked on tryptose soya agar containing 5% defibrinated sheeps' blood and amphotericin B, vancomycin, and cefaperazone [40]. Plates were incubated for 48 hours at 37°C in an airtight container with a Campy *Gen *sachet (Oxoid, Basingstoke, United Kingdom) and scored for growth of *C. jejuni*. Infected mice were necropsied 30 days after inoculation and *C. jejuni *populations recovered from the cecum. For subsequent passages, the inoculum was prepared using pooled *C. jejuni *populations from the ceca of the mice in the previous passage after confirmation that no contaminants were present. Each strain was used to inoculate five mice in the second and third passages and ten mice in fourth and final passage. Four mice in the first passage, five mice in the second and third passages, and ten mice in the final passage were sham inoculated with tryptose soya broth to serve as controls.

For all experiments, 8–12 week old mice were infected with ~1 × 10^10 ^cfu *C. jejuni *by oral gavage and observed daily for clinical signs. Mice were euthanized and necropsied promptly when clinical signs of disease developed or at thirty days post-infection. Blood samples were obtained by cardiac puncture after death. Observations on gross pathological changes were recorded during necropsy. Tissue snips from stomach, jejunum, cecum, and colon were spread on agar plates selective for *C. jejuni *(tryptose soya agar plates with 5% sheeps' blood and cefaperazone, amphotericin B, and vancomycin (TSA-CVA) [40]). All of the *C. jejuni *growth from cecal tissue of each individual mouse was harvested from the agar surface and frozen at -80°C to be used as the inoculum for the next serial passage. To produce the inoculum for the next passage, each frozen culture was spread on a tryptose soya sheeps' blood agar plate with no antibiotics and incubated for 24 hours at 37°C under a 10:10:80 mixture of H_2_, CO_2_, and N_2_; this growth was used to inoculate a second plate which was incubated 12 hours as before. Growth from the second plate was suspended in broth, and purity and motility were verified by light microscopy and Gram staining. The suspension was adjusted to an OD_600 _of 1.0; the growth from all plates of a single strain was pooled to produce the inoculum. Aliquots of each inoculum were suspended in tryptose soya broth containing 15% glycerol and stored at -80°C for further studies.

In the first serial passage, mice were inadvertently shifted from the diet containing an ~12% minimum fat to a diet containing an ~6% minimum fat just prior to inoculation with *C. jejuni*. This error was not discovered until after the mice had been inoculated. A previous experiment with *C. jejuni *infected mice on the ~12% fat diet and ~6% fat diets did not reveal a statistically significant difference in survival, gross pathology, or histopathology scores. Therefore, all subsequent passages included a similar dietary shift. In an experiment conducted in parallel with the final passage, 10 mice on the ~12% fat diet and 10 mice that had experienced the dietary shift were inoculated with non-adapted (unpassaged) *C. jejuni *11168. That experiment did show a statistically significant difference in histopathology scores in mice on these two diets, so a third comparison of diets was done to try to resolve the issue. Nineteen mice each were kept on the ~12% fat diet, shifted onto the ~6% fat diet at least two weeks prior to the experiment, or subjected to the ~12% fat to 6% fat diet transition 3 to 5 days prior to inoculation as experienced by the mice in the serial passage experiment. Ten mice in each of the three diet groups were inoculated with non-adapted *C. jejuni *11168 and nine mice on each diet regime were inoculated with tryptose soya broth as controls.

Finally, we conducted a short-term experiment to determine whether there were differences in events in early infection between the original and mouse-adapted *C. jejuni *11168 strains. Ten mice each were inoculated with unpassaged *C. jejuni *11168, inoculum prepared from the *C. jejuni *11168 culture used to inoculate the mice in the fourth (final) passage, or tryptose soya broth. All mice were kept on the ~12% fat diet throughout this experiment and were necropsied 48 hours after inoculation.

### Enzyme-linked immunosorbant (ELISA) assays

Plasma samples were assayed for *C. jejuni*-specific antibodies as previously described [40] using antigen prepared from non-adapted *C. jejuni *11168.

### Histopathology

Hematoxylin and eosin stained sections of the ileocecocolic junction of each mouse were scored as described previously on a scale of 0 to 44 [40]. For non-parametric statistical analysis, this scale was divided into grades of 0 (scores of 0 to 9), 1 (scores of 10 to 19), and 2 (scores of 20 to 44).

### Statistical analysis

Cluster analysis based on DNA sequences of housekeeping loci of the *C. jejuni *strains utilized sequence data from the *Campylobacter jejuni *Multi Locus Sequence Typing website http://pubmlst.org/campylobacter/[7] and data generated in our laboratory for strain NW. Alignment and clustering were performed with ClustalW2 http://www.ebi.ac.uk/Tools/clustalw/index.html#[70] using default parameters. Reference strains established by Wareing et al. [42] were also included. Clustering analysis of manually scored RFLP patterns was performed using the Cluster V0.1 calculator http://www2.biology.ualberta.ca/jbrzusto/cluster.php developed by John Brzustowski [71]. The Jaccard similarity coefficient and the Saitou and Nei neighbor-joining clustering method were used.

Fisher's exact test and the Freeman Halton extension of Fisher's exact test were performed using the VassarStats calculator http://faculty.vassar.edu/lowry/VassarStats.html[72].

Kaplan Meier log rank survival analyses were performed using SigmaStat 3.1 (Systat Software, Port Richmond, CA).

Gross pathology, histopathology, and ELISA data were analyzed using SigmaStat 3.1. The nonparametric Kruskal Wallis one-way ANOVA was used for gross pathology and histopathology scores in the serial passage experiment. Scores for analysis of gross pathology data were assigned as follows: no gross pathology, 1; either enlarged ileocecocolic lymph nodes or thickened colon wall, 2; both enlarged ileocecocolic lymph nodes and thickened colon wall, 3; enlarged ileocecocolic lymph nodes, thickened colon wall, and bloody contents in lumen, 4. Kruskal Wallis nonparametric one-way ANOVA was performed on these scores; if a significant result was obtained, post hoc comparisons were made using Fisher's exact test. For this test, the two-way table was cast so that mice with no gross pathology (score of 1) were compared to mice having all levels of gross pathology (scores 2, 3, and 4) combined; correction for multiple comparisons was done using the Holm-Šidák procedure [73]. Histopathology scores were analyzed as previously described [40]. Two-way ANOVA and post hoc tests with correction for multiple comparisons were used for gross pathology and histopathology scores in the diet comparison experiment; because scores were not normally distributed, the analysis was performed on ranks rather than raw scores.

Histopathology scores from the serial passage experiment were further analyzed using the Mantel test for trends with correction for continuity [49]; for this test, data were cast in a two-way table for each *C. jejuni *strain according to the number of the serial passage of the strain and the number of animals exhibiting lesions of grades 0 and 1 combined (scores ≤ 19) compared to the number of animals exhibiting lesions of grade 2 (scores ≥ 20). The choice to divide the data in this way for this analysis was made because almost all of the medians of the histopathology scores of C57BL/6 IL-10^-/-^mice infected with non-adapted *C. jejuni *11168 for 28–35 days in previous experiments fell into grade 1 (median scores between 9.5 and 19; [40] and unpublished data), whereas the median scores of mice infected with serially passaged *C. jejuni *11168 all fell into grade 2.

ELISA data were transformed as previously described [40] prior to analysis by one or two-way ANOVA with post hoc tests using SigmaStat 3.1.

GACK analysis was performed on the microarray data using programs available at http://falkow.stanford.edu/whatwedo/software/software.html[52].

## Authors' contributions

JAB participated in preparation of the paper, developing the experimental design, and overseeing the experiments and performed the following experimental work: preparation of *C. jejuni *inocula, culture of *C. jejuni *from mouse tissues, and scoring of culture results. JLS conducted the RFLP analysis of virulence genes. AJM conducted culture of *C. jejuni *from mouse tissues, DNA isolation from mouse tissues for *C. jejuni*-specific PCR, confirmatory PCR for *C. jejuni*, MLST typing of strain NW, and ELISA. VAR conducted blood and tissue harvesting from mice at necropsy. AEPJ, ELS and LSM participated in development of the *C. jejuni *whole ORF microarray, and AEPJ performed the hybridizations. JEW analyzed the microarray data. JRG confirmed the IL-10 genotype in the mice by PCR and performed PCR amplification and sequencing of ORFs Cj0874c and Cj0987c. TSW performed an initial analysis of published MLST data that guided the choice of strains to be studied. LSM gained funding for the projects, participated in developing the experimental design, preparing the paper, and overseeing the experiments, and performed the following experimental work: clinical assessments of mice, blood and tissue harvesting from mice at necropsy and evaluation of hematoxylin and eosin stained tissue sections.

## Supplementary Material

Additional file 1 **Table S1**. *C. jejuni *colonization status of mice at necropsy. The data provided show the percent of mice in which *C. jejuni *was detected at necropsy by culture or by PCR assay in feces and four sites in the gastrointestinal tract in experiments reported in the main text. **Table S2**. Genes present in strain 11168 but confirmed absent or strongly divergent in strain NW. The data provided show all genes present in strain 11168 but absent in strain NW that were detected by microarray analysis and confirmed by PCR assay and their functions as noted in the most recent annotation of the *C. jejuni *11168 genome [53]. Click here for file
