# Supplementary material for: Multiple factors interact to produce responses resembling spectrum of human disease in Campylobacter jejuni infected C57BL/6 IL-10-/- mice
Source: BMC Microbiol. 2009 Mar 18;9:57. doi: 10.1186/1471-2180-9-57 (PMC2669091; doi:10.1186/1471-2180-9-57)
Supplement: Additional file 1 — Table S1. C. jejuni colonization status of mice at necropsy. The data provided show the percent of mice in which C. jejuni was detected at necropsy by culture or by PCR assay in feces and four sites in the gastrointestinal tract in experiments reported in the main text. Table S2. Genes present in strain 11168 but confirmed absent or strongly divergent in strain NW. The data provided show all genes present in strain 11168 but absent in strain NW that were detected by microarray analysis and confirmed by PCR assay and their functions as noted in the most recent annotation of the C. jejuni 11168 genome [53]. [file 1471-2180-9-57-S1.pdf]

Table S1. *C. jejuni* colonization status of mice at necropsy.

				% of mice positive by culture (% of mice negative by culture but positive by PCR assay)*				
**Strain**	**Passage (diet)**	***n***	**Dose (cfu)**	**Stomach**	**Jejunum**	**Cecum**	**Colon**	**Feces**
**A. Serial passage adaptation experiment**								
**TSB**	all (12 to 6)	29	0	0	0	0 (0)	0	0
**11168**	1 (12 to 6)	5	2.9×10^{10}	40 (0)	60 (0)	100	80 (0)	100
	2 (12 to 6)	5	6.8×10^{9}	60 (0)	100	100	100	100
	3 (12 to 6)	5	1.8×10^{10}	100	100	100	100	100
	4 (12 to 6)	10	1.3×10^{10}	70 (30)	100	100	100	100
**D0835**	1 (12 to 6)	5	1.8×10^{10}	60 (20)	40 (0)	100	100	100
	2 (12 to 6)	5	1.8×10^{10}	20 (20)	100	100	100	100
	3 (12 to 6)	5	8.2×10^{9}	20 (0)	100	100	100	100
	4 (12 to 6)	10	1.1×10^{10}	70 (0)	90 (10)	100	100	100
**D2586**	1 (12 to 6)	5	1.0×10^{10}	60 (0)	40 (0)	100	100	100
	2 (12 to 6)	5	7.2×10^{9}	60 (0)	80 (0)	100	100	100
	3 (12 to 6)	5	1.3×10^{10}	40 (0)	60 (0)	100	100	100
	4 (12 to 6)	10	1.4×10^{10}	60 (10)	70 (0)	100	90 (0)	100
**D2600**	1 (12 to 6)	5	1.0×10^{10}	40 (0)	40 (20)	100	100	100
	2 (12 to 6)	5	9.0×10^{9}	40 (0)	60 (0)	100	100	80 (0)
	3 (12 to 6)	5	1.5×10^{10}	40 (20)	60 (0)	100	100	100
	4 (12 to 6)	10	8.8×10^{9}	80 (20)	100	100	100	100
**NW**	1 (12 to 6)	5	1.9×10^{10}	40 (0)	40 (0)	100	100	100
	2 (12 to 6)	5	1.4×10^{10}	60 (20)	80 (0)	100	100	100
	3 (12 to 6)	5	9.2×10^{9}	60 (40)	80 (20)	100	100	100
	4 (12 to 6)	9	1.0×10^{10}	70 (30)	80 (10)	100	100	100
**D0121**	1 (12 to 6)	5	2.2×10^{10}	0 (0)	0 (20)	0 (20)	0 (0)	0 (0)
**33560**	1 (12 to 6)	5	1.6×10^{10}	0 (80)	0 (100)	0 (60)	0 (100)	0 (0)
**11168**	unpassaged (12)	10	1.6×10^{10}	50 (20)	50 (40)	100	100	90 (0)
**11168**	unpassaged (12 to 6)	10	1.6×10^{10}	70 (0)	70 (10)	100	100	100
**B. Follow-up diet comparison experiment; non-adapted strain 11168**								
**% fat in diet**								
**12**	unpassaged 11168	10	1.9×10^{10}	60 (10)	60 (0)	100	100	100
	TSB	9	0	0	0	0	0 (0)	0
**12 to 6**	unpassaged 11168	10	1.9×10^{10}	60 (20)	100	100	100	100
	TSB	9	0	0	0	0	0 (0)	0
**6**	unpassaged 11168	10	1.9×10^{10}	40 (40)	80 (10)	100	100	100
	TSB	9	0	0	0	0	0 (0)	0
**C. Short term infection experiment**								
**Inoculum**								
**TSB**	control	10	0	0	0	0	0	0
**11168**	unpassaged	10	1.7×10^{10}	100	80 (0)	100	100	100
**11168**	passaged	10	1.7×10^{10}	100	100	100	100	100

* Cultured isolates were verified by *C. jejuni*-specific PCR assay. PCR was only performed on tissues of infected mice for which the corresponding culture was negative. PCR was only performed on cecal tissue of TSB-inoculated mice. *C. jejuni* was not detected by culture or PCR assay in any of 29 sham-inoculated mice in the passage experiments.

Table S2. Genes present in strain 11168 but confirmed absent or strongly divergent in strain NW

Gene	Function, putative function, or similarity*	Gene	Function, putative function, or similarity*
**O-linked glycosylation (flagellin) locus**		**LOS synthesis**	
Cj1296	efflux pump	*gmh*A	lipooligosaccharide biosynthesis gene
Cj1297	efflux pump	**Periplasmic proteins**	
Cj1301	unknown	Cj0424	putative acidic periplasmic protein
Cj1321	putative transferase	Cj0265c	cytochrome C-type heme-binding protein
Cj1323	hypothetical protein	*mod*A	periplasmic protein; molybdate transporter
Cj1324	resembles *P.aeruginosa* LPS gene	Cj1723c	putative periplasmic protein
Cj1325	methyltransferase	**Restriction and modification**	
Cj1326	hypothetical protein	Cj1549c	R subunit protein
**Capsule locus**		Cj1551c	S protein
Cj1417c	resembles glutamine amidotransferase class-I	Cj1553c	Type I N-6 DNA methylase
Cj1418c	resembles PEP synthase	**Other functions**	
Cj1419c	possible methyltransferase	*acp*P4	possible acyl carrier protein
Cj1420c	resembles methyltransferase	*bio*C	possible biotin synthesis protein
Cj1422c	possible sugar transferase, MIP family signature	Cj0055c	copper amine oxidase
Cj1426c	possible methyltransferase	Cj0302c	resembles molybdenum recognition protein
Cj1427c	possible NAD dependent epimerase/dehydratase	Cj1552c	hypothetical ATPase
Cj1429c	hypothetical protein	*fcl*	probable fucose synthetase
Cj1430c	possible dTDP-4-dehydrorhamnose 3,5-epimerase	*kfi*D	probable UDP-glucose 6-dehydrogenase
Cj1434c	putative sugar transferase	**Hypothetical proteins**	
Cj1435c	HAD-superfamily protein	Cj0008	hypothetical protein
**Membrane proteins**		Cj0565	uncharacterised protein family
Cj0260c	two probable transmembrane helices	Cj0566	hypothetical protein
Cj0423	two probable transmembrane helices	Cj0814	hypothetical protein
Cj0860	ten probable transmembrane helices	Cj0815	hypothetical protein
Cj1484c	putative membrane protein	Cj0816	hypothetical protein
Cj1721c	possible outer membrane protein	Cj0859c	hypothetical protein
Cj0629	possible lipoprotein	Cj0970	hypothetical protein
Cj0818	putative lipoprotein	Cj1144c	hypothetical protein
Cj1677	putative lipoprotein	Cj1306c	hypothetical protein (617 family)
Cj1678	possible lipoprotein	Cj1679	hypothetical protein, contains tetratricopeptide

* From the re-annotation of the *C. jejuni* 11168 genome by Gundogdu et al. [54].
